# Mapping the flow of commercial broiler day-old chicks in Kenya

**DOI:** 10.3389/fvets.2025.1607825

**Published:** 2025-12-04

**Authors:** Eugine L. Ibayi, Jane N. Nyambura, Boru A. Guyo, Arshnee Moodley, Dishon M. Muloi

**Affiliations:** 1 Health Program, International Livestock Research Institute, Nairobi, Kenya; 2 Department of Veterinary and Animal Sciences, University of Copenhagen, Frederiksberg, Denmark; 3 Institute of Infection, Veterinary and Ecological Sciences, University of Liverpool, Liverpool, United Kingdom

**Keywords:** broiler, day-old chick, breeding, antibiotic use, value chain, Kenya

## Abstract

**Introduction:**

Poultry production is a significant contributor to economic growth and food security in Kenya. Reliable data on day-old chicks (DOCs) production and distribution are essential for formulating effective national policies on poultry production and animal health delivery. Here, we describe the commercial broiler DOCs production, distribution, and associated animal health and antibiotic use practices in Kenya.

**Methods:**

Data were collected through focus group discussions, individual interviews, and key informant interviews involving three DOCs producers, 128 distributors, two government regulatory agencies, one poultry producers’ association and 128 farmers.

**Results:**

Across the DOCs production pyramid, animal health and biosecurity practices varied, with stronger management observed in parent stock (PS) than in commercial stock (CS) production facilities. DOCs distribution followed either vertically integrated systems for largescale broiler farms—owned by the CS producers or contracted farms—or horizontal systems involving numerous small-scale distributors. Veterinary drug stores were the main distributors (96%) of CS DOCs to farmers. Veterinary authorities routinely audited PS and CS hatcheries for compliance with biosecurity and animal welfare standards. However, suboptimal animal welfare practices (such as long travel times and transportation stress) and inappropriate antibiotic recommendation to farmers were observed at the distribution level during collection of CS DOCs.

**Discussion:**

These findings provide critical evidence to guide policies on biosecurity, distribution, and animal health practices within the DOCs distribution chain. Factors such as transportation stress, poor biosecurity, and inappropriate antibiotic recommendations can compromise DOCs health, which may result in higher antibiotic use on farms. Strengthening oversight and promoting best practices across the production and distribution system would enhance the quality and health of DOCs supplied to farmers, reduce dependence on antibiotics, and support sustainable poultry production that safeguards both food security and public health.

## Introduction

The global poultry production is expanding rapidly, with chicken meat production rising from 94 million tons in 2012 to 123.6 million tons in 2022 (1). This growth is particularly evident in low- and middle-income countries, driven by rising demand for livestock products associated with rise in incomes, urbanization and population growth (2, 3).

Poultry farming is the most common form of livestock keeping in Kenya, with estimates from 2022 indicating that about 75% of rural and peri-urban households keep chickens for income generation and household food security (4–6). The sector contributes approximately 30% of the agricultural gross domestic product and 8% to the national gross domestic product (7). Within the poultry sector, broiler production represents about 22% of the national poultry population in the country and continues to expand particularly in urban and peri-urban areas. For example, in Nairobi city, the capital of Kenya, chicken consumption is projected to increase fivefold from six metric tons in 2000 to 30 metric tons by 2030 (8).

Globally, commercial broiler farming operates through a highly structured breeding pyramid, with genetic stock (great-grandparent, grandparent (GP), and parent stock (PS)) at the top supplying the global broiler industry (9, 10). Over recent decades, consolidation within the pedigree breeding sector has resulted in most commercial stock (CS) day-old chicks (DOCs) originating from a limited number of nucleus breeding companies selected for specific performance traits (10, 11). This highly agglomerated hatchery supply chains present opportunities for small and medium scale farmers to access high-quality, fast-growing poultry breeds but also poses risk of vertical spread of pathogens and antimicrobial resistance due to their intensive production systems and complex distribution channels. During the hatchery production stage and distribution, DOCs are highly susceptible to pathogen transmission, either vertically or through the hatchery and distribution environment eventually reaching farms (12–15). Suboptimal hatchery welfare and nutrition, poor hatching conditions, or inadequate biosecurity can result in compromised DOCs quality and high infection likelihood downstream (16, 17). Farmers receiving such DOCs may consequently depend more on antibiotics to maintain flock health and productivity. Understanding the production and distribution channels of DOCs, along with the associated animal health and disease control practices, is therefore critical to inform targeted interventions that enhance animal health, welfare and stewardship. Here, we use a value-chain mapping framework to characterize the distribution network of commercial broiler DOCs and the associated animal health and antibiotic use practices across the supply chain in Kenya.

## Materials and methods

### Study area and design

**Figure 1:**
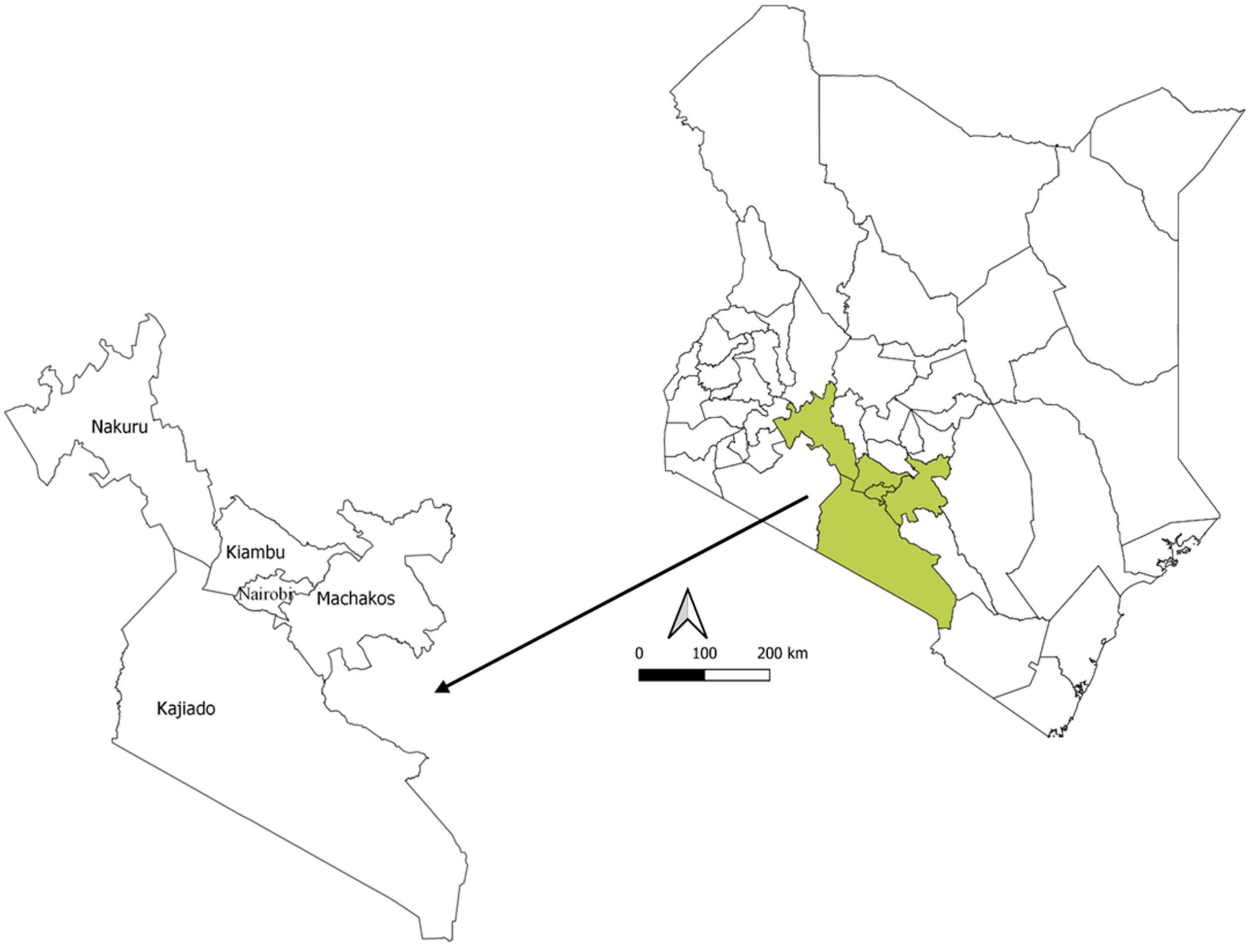
Map of Kenya with the study areas highlighted in green.

We conducted a cross-sectional, qualitative mixed-methods study. The study began with a literature review to develop an overview of broiler DOCs production and distribution in Kenya. Six types of stakeholders (breeders and hatcheries, distributors, regulators, associations, and farmers) along the production pyramid and distribution channels were identified and recruited (Table 1). These stakeholders were located in five geographical areas (Nairobi, Kiambu, Kajiado, Machakos, and Nakuru counties), which represent the main poultry production areas in Kenya (9) (Figure 1). A formal sample size calculation was not undertaken, as the study adopted an exploratory qualitative design. This approach aimed to capture perspectives from diverse stakeholder groups across the production and distribution system and to reach thematic saturation—the point at which no new insights emerged from additional interviews. It aligns with established qualitative research methodology, particularly for studies seeking to understand structures, practices, and decision-making processes within agri-food and health systems (18).

**Table 1:** List of stakeholders who participated in the study.

Interview type	Description of stakeholder	Number interviewed
Focus group discussion	Day-old chicks distributors	30
Key individual interview	Veterinary regulator	1
	Poultry associations	2
Individual interviews	Day-old chicks’ distributors	128
	Commercial stock producers	2
	Parent stock producers	1
	Broiler farmers	128
Total		292

Data were collected through three approaches: focus group discussions (FGD), key informant interviews (KII), and individual interviews. Participants were carefully selected from each node along the distribution channel to ensure representation of the different stakeholders. A total of 128 farmers, separately 128 distributors (including hatchery agents (outlets/individuals contracted by hatcheries to aid with distribution of DOCs) and veterinary drug stores (outlets that stock, sell, and distribute farm and veterinary input including DOCs)), two regulators (directorate of veterinary services and directorate of livestock production), one poultry producers’ association, two CS producers, and one PS producer were recruited and interviewed. Six FGDs were conducted, each comprising 4–15 participants to allow meaningful engagement. Hatcheries were identified through an initial literature review and discussions with government officials.

Distributors were identified via a cross-sectional survey in major towns across three counties: Nairobi, Kajiado, and Machakos, where veterinary drug stores and hatchery agents were selected and recruited. In the absence of a registry of veterinary drug stores and hatchery agents, it was not possible to randomly sample distributors in each town, so convenience sampling was used, in addition to a snowballing approach where new distributors were recommended by fellow distributors. Key informants were recruited based on their expertise and knowledge of the overall distribution channel. The study was done between June 2023 to January 2024, a period spanning both the dry and wet seasons in Kenya, thereby capturing seasonal fluctuations that may influence DOCs flow and associated management practices. The final distribution is provided in Table 1.

### Data collection

During FGDs with distributors, interview guides were used to facilitate the discussions, and all proceedings were audio recorded. The FGD guides covered the following themes: (1) the role of the participants; (2) the structure and description of the production or distribution chain; (3) interactions with other stakeholders and regulatory topics such as policies and regulations; and (4) practices and perceptions related to animal health and antibiotic use. Open-ended prompts, such as “why” and “how,” were used to facilitate engagement amongst participants until a consensus was reached.

In addition to the FGD, individual interviews were administered to all distributors using a structured questionnaire to collect data on (1) source of DOCs; (2) vaccination history; (3) quality check of the DOCs on reception; (4) purchase and sale process. To complement the data gathered in the FGD and individual interviews, KII were conducted with individuals from regulatory authorities and poultry associations. KII participants were considered stakeholders with first-hand knowledge of the CS DOC production pyramid, distribution channels, and governance structures. A similar approach was used in the KII as in the FGD, with the addition of drawing flowchart diagrams to visualize and describe the stakeholders involved and major nodes in the distribution chain.

All interviews were conducted by trained researchers in both English and Swahili, with Swahili transcripts subsequently translated into English. Random back-checks of translations were performed by the research supervisor to ensure accuracy and consistency. Interviewers received prior training on research ethics, probing techniques, and consistency in notetaking. Data collection tools were pretested and refined before use in the field. Field supervisor routinely reviewed completed transcripts and audio recordings to ensure completeness and internal consistency. All the research data were stored in password-protected server accessible only to the research team.

### Data analysis

Audio recordings from FGD with distributors were carefully listened to and transcribed into structured templates. Flow diagrams developed during FGD and KII discussions were included into these templates. Data analysis included two parts. First, information from the flowchart exercises was used to generate a detailed flowchart diagram using BioRender software (19), illustrating the stakeholders involved in the distribution chain and mapping the flow of DOCs from the source hatcheries through distributors to farmers. Second, data extracted from the transcript templates were systematically organized and entered into Microsoft Excel together with quantitative data from individual interviews. These were categorized into three main themes: (1) sourcing and transportation of DOCs, (2) assessment of DOC quality during purchase and distribution, and (3) sale processes. Thematic patterns emerging across these activities were synthesized and presented narratively (20).

## Results

### Broiler production pyramid

**Figure 2:**
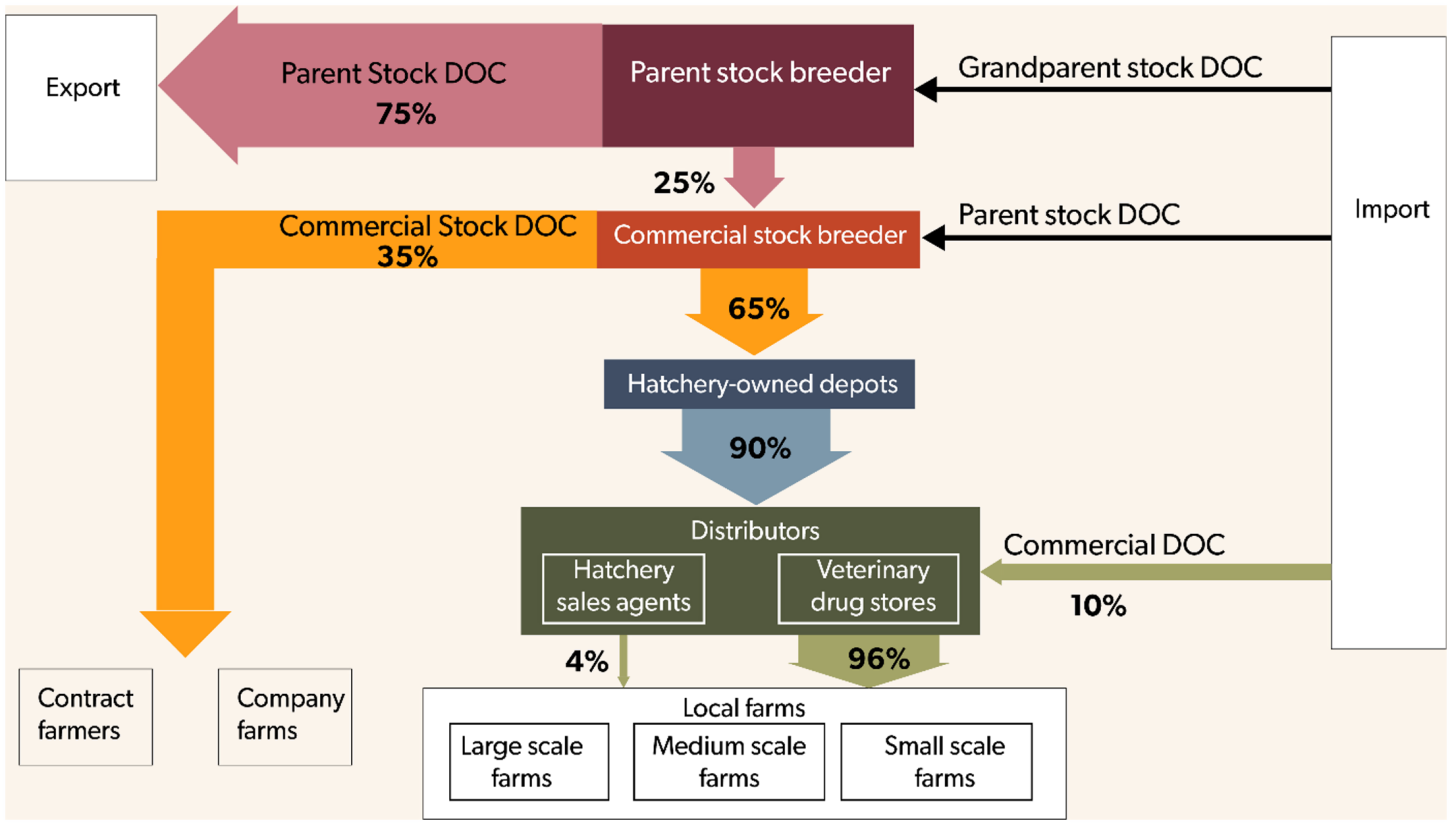
The production pyramid for commercial broilers in Kenya and the actual flow of commercial broiler day-old chicks within the country. The arrows indicate the directional flow of day-old chicks through the production and distribution chain.

The global broiler production pyramid typically comprises five hierarchical levels: pedigree pure line stock, great-grandparent stock, GP, PS, and CS. In Kenya, however, we found that the pyramid is truncated, consisting only of the GP, PS, and CS, as the pedigree and great-grandparent levels are absent in the country. Kenya has a single GP farm, which imports GP DOCs of the Hubbard breed from Europe. Interviews with the single GP indicated that it supplies approximately 25% of its DOCs to PS farms within the country and exports the remaining 75% to other African countries (Figure 2). Data from veterinary authorities indicated that Kenya has 14 PS farms that supply CS DOCs locally. These PS farms supply 90% of the total CS DOCs market share, with the remaining 10% being imported. CS hatcheries are typically integrated operations (*n* = 2), producing DOCs for their own farms while also supplying farms across the country.

### Flow and distribution of commercial broilers

The distribution channel of CS DOCs is illustrated in Figure 2 and involves four main stakeholders: CS hatcheries, hatchery-owned depots, distributors, and farmers. Information from interviews indicate that approximately 35% of DOCs produced by CS hatcheries are supplied directly to their own farms or contracted farms through vertically integrated systems, while about 65% are channeled to hatchery owned depots. The depots are strategically located in major towns across the country, serving as intermediaries between hatcheries and downstream distributors—mainly veterinary drug stores and hatchery agents. Veterinary drug stores were identified as the main distributor group, handling about 96% of all CS DOCs sourced from depots, whereas hatchery agents accounted for the remaining 4%. Both groups sell DOCs directly to farmers. Discussions with farmers and KIIs revealed that the local DOCs supply chain constitute about 90% of the national market share, while the remaining 10% is imported from neighboring Uganda. These proportions were reported to fluctuate seasonally, with imports increasing during peak months of November and December, as local supply was described as relatively stable throughout the year. In addition to broiler DOCs, 50% of distributors also supplied indigenous chicken DOCs, 44% supplied layer DOCs, and 6% supplied ducklings.

We found that hatcheries transported DOCs to depots and distributors using modified vehicles designed for safe chick transport. Most farmers (84%) reported using vehicles to transport DOCs, while 9.1% used motorbikes and 6.8% relied on public transport.

### Practices at the breeding farms and hatchery, and distribution levels

#### Breeding farm and hatchery practices

Breeding farms (PS and CS producers) and hatcheries reported strict adherence to biosecurity and vaccination protocols. GP flocks were routinely vaccinated against Newcastle disease, infectious bronchitis, *Salmonella*, infectious bursal disease, and Marek’s disease. Some CS producers vaccinated DOCs against Newcastle disease, Infectious Bronchitis, and Infectious Bursal Disease, on newly hatched DOCs.

During production in the breeding farms, the GP DOCs were segregated by sex and reared in dedicated sections for the first 15 weeks to ensure uniformity and to achieve target weights. At 16 weeks, they are transferred to breeding units where males and females are mixed for reproduction. This breeding phase continues until the 54th week, at which point the flock is culled. The primary objective during this stage is production of fertile eggs (hatching eggs). Fertile eggs go through a rigorous sorting process, with only those meeting quality standards for cleanliness, size, shape, and shell integrity selected for production. Eggs were stored in cold rooms for up to 3 weeks before incubation, as prolonged storage reduced hatchability. Incubation schedules were planned according to pre-orders.

In all visited hatcheries, the facility designs prioritized hygiene and biosecurity. Functional separation of egg receiving, incubation, hatching, and chick-handling areas minimized cross-contamination, reinforced by a unidirectional workflow from egg intake to DOCs dispatch. Additionally, hatcheries had automated incubators, where temperature, humidity, and egg-turning (takes place at 45° angle) were controlled to ensure the best embryo development. After hatching, DOC quality was evaluated by trained staff for navel condition, activity, and reflex response before vaccination and packaging into ventilated boxes to ensure adequate airflow and warmth during transport. Detailed records on egg sources, incubation conditions, vaccination history, and DOCs distribution were maintained to support traceability in case of disease outbreaks.

#### Distribution nodes

Of the 128 distributors interviewed, 75% were female, with most (57%) aged between 28 and 37 years, and 21% lacking any form of animal health training. Vaccination history against common diseases, Marek’s and Gumboro, the availability and reliability of the distributor, and farmer preferences for the DOCs producer were reported by 34, 28, and 20% of distributors, respectively, as the main factors in deciding which hatchery to purchase DOCs from.

Other factors included reputation of the company (5.3%), affordability of the DOCs (4.9%), provision of after-sale services (3.2%), recommendation by other distributors (2.8%). One quarter (24%) of distributors repackaged DOCs into different boxes, primarily due to customer demand (83%), with the remaining repackaging done to replace mortalities (14%) and generate profit (3%). Before delivering DOCs to farms, 25% of distributors provided either multivitamins or glucose (25% each) dissolved in water, only water (18.7%), liquid paraffin or antibiotics (12.5% each), or watermelon (6.3%) to the DOCs. Seventy-three percent of the distributors provided after-sale services to farmers, mostly animal health advice (64%) and delivery of DOCs to the farms (36%). Distributors self-reported implementing various biosecurity measures when delivering DOCs to farms, with the most common practices including disinfecting delivery vehicles between farms (38%), avoiding entry into farms (33%), changing personal protective equipment between deliveries (25%), and avoiding entry into the chicken house (4%).

Before accepting DOCs from the hatcheries, distributors assessed several quality parameters, including vaccination history (43%), activity of the DOCs (30%), physical conformation (14%) and health appearance (9%). Other considerations were viability (2%), the numbers delivered (1%) and size of the DOCs (1%). Verification of vaccination status of CS DOCs varied. Most distributors relied on labels on the DOCs transportation boxes (50.0%), word of mouth (26.0%), and vaccination certificates (14.0%). Other methods were writing on the receipts (5.0%), changing fecal color or blue marking on the neck (3.3%), and trusting the company without verification (1.7%).

Distributors reported that they verified that DOCs had been vaccinated against Marek’s disease (44%), Newcastle disease (30%), and Infectious Bursal disease (Gumboro) (22%), and Infectious Bronchitis disease (4%). Most distributors (94%) sold all the DOCs within a day, while the remainder sold them within two or more days. Distribution typically occurred during cooler morning hours or evening to reduce heat stress.

Overall, 25.8% of distributors reported losses of up to 5% of DOCs occurring between collection from hatcheries and delivery to farms. The main causes of death were transportation stress and injuries (38%), infections or indigestion (19%), and poor brooder management (12%) (Table 2). To minimize mortality, distributors maintained clean, well-ventilated spaces (49%), monitored temperature and sunlight exposure (26%), and prevented overcrowding (16%). Additional measures employed by the distributors included administering antibiotics (3.5%), immediate dispatch (2.5%), and providing multivitamins, close monitoring, careful handling, and rehydration (1% each).

**Table 2:** Reported causes of mortality in commercial day-old chicks, as stated by distributors, from the time they leave the hatchery until the end of their first week at local commercial broiler farms.

Characteristic	*N* = 473 (%)
Transportation stress and injuries, e.g., extremes in humidity and temperature	179 (38%)
Infection and indigestion	88 (19%)
Poor management in the brooder, e.g., high stocking density, poor ventilation, lack of starter pack	56 (12%)
Poor day-old chicks’ handling and poor farm management	42 (8.9%)
Hatchery problem (i.e., yolk sac infection and poor-quality chicks)	40 (8.5%)
Poor biosecurity at the distribution point and farm	13 (2.7%)
Poor packaging	3 (0.6%)
Poor quality feeds	2 (0.4%)

#### Broiler farm practices

We interviewed 128 broiler farmers, 81% of whom were female. More than half (58%) sourced their DOCs from veterinary drug stores, 36% obtained them from hatchery sales agents, and 6% imported DOCs from Uganda. 82% of farmers reported being aware that the DOCs they acquired had been vaccinated, with 44.1% of those unaware of the specific vaccines administered. Most farmers (86%) relied on distributors for vaccination information, 10% assumed vaccination based on hatchery practices, and 4% verified it through certificates.

#### Antibiotic use practices

**Figure 3:**
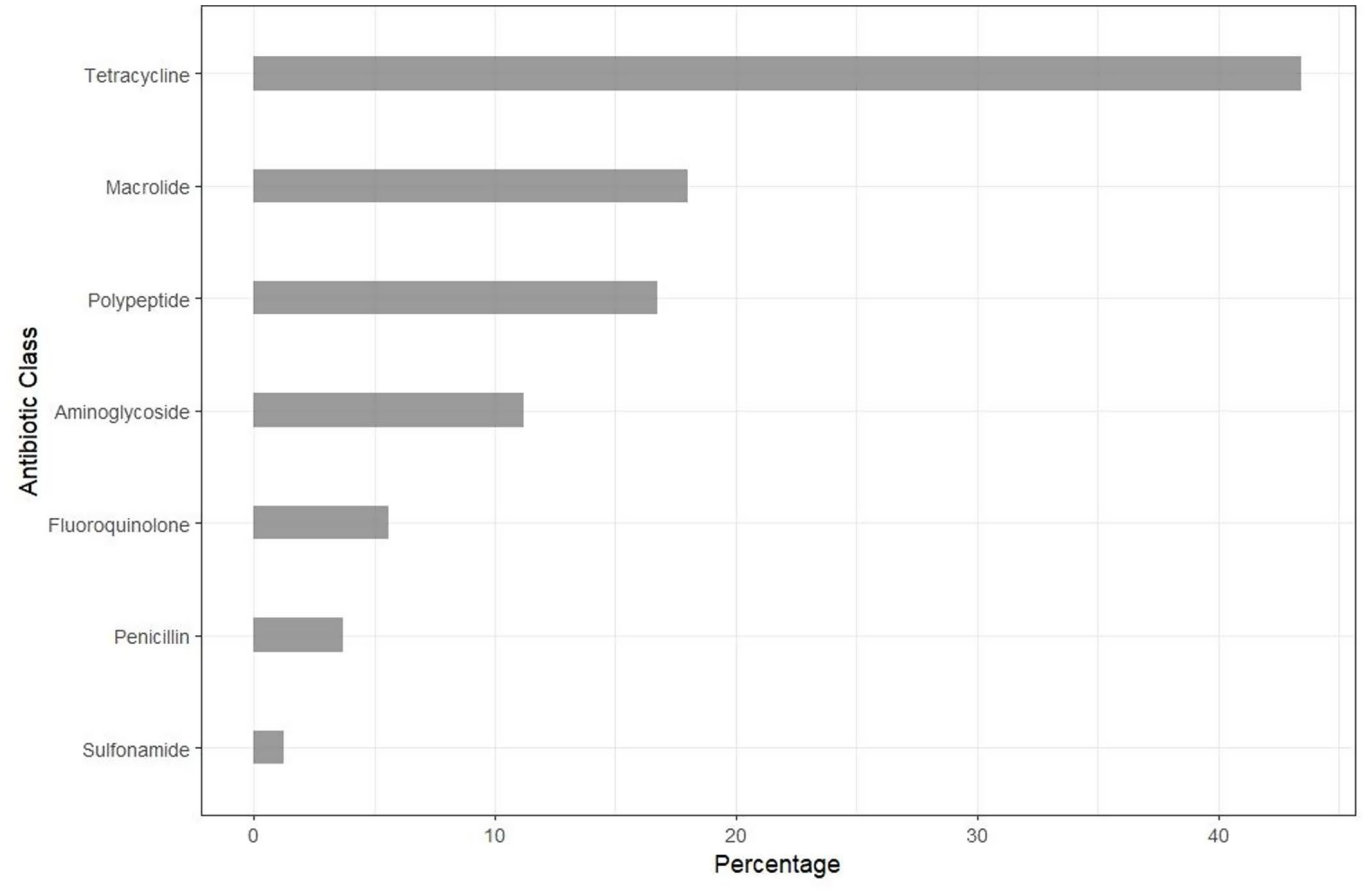
Antibiotic classes recommended by distributors to commercial broiler farmers at the time of commercial day-old chicks’ collection.

Hatcheries reported that they did not administer antibiotics to DOCs, but 3.5% of distributors indicated that they administered antibiotics to DOCs while on their premises. Half (54%) of distributors reported that they recommended antibiotics to broiler farmers during the DOCs purchase process. The reasons provided by distributors for recommending antibiotics to farmers included helping farmers prevent disease outbreaks and mortality on farms (59%), meeting farmers’ expectations (30%), increasing sales (7%), “boosting DOCs immunity” (2%), and relieving stress (2%). The three most recommended antibiotic classes by distributors were tetracycline, macrolides, and polypeptides (43.5%), 18.0 and 16.8%, respectively, (Figure 3). Alisery WS (Interchemie, Holland), a brand with four different active pharmaceutical ingredients namely, erythromycin (macrolide), streptomycin (aminoglycoside), oxytetracycline (tetracycline), and colistin (polymyxin B) was the most common reported brand.

## Discussion

This study mapped the production and distribution channels of commercial broiler DOCs, identified the key stakeholders involved, and described associated animal health management and antibiotic use practices.

The Kenyan broiler production pyramid begins at the PS level, with only one farm in the country. This farm imports grandparent stock DOCs, but only a quarter of the DOCs produced are distributed locally, leading to a heavy reliance on imports for most parent stock DOCs. First, while this system allows smallholder and medium-scale poultry producers to access high-quality, fast-growing breeds, it also increases production costs due to import dependence, thereby constraining profitability for small-scale farmers (21). Second, at the global level, the hierarchical structure of the chicken breeding industry has facilitated both the maintenance and transmission of poultry and zoonotic pathogens including AMR strains, such as *Salmonella* and extended spectrum beta-lactamase producing *E. coli* (22). These organisms can be vertically transmitted across breeding and production chains raising concerns about the potential introduction and spread of foreign infectious agents through international poultry trade (23). Third, the imported DOCs are typically from an environment different from local conditions, but well suited in the countries of origin. Therefore, the chicken may take considerable time to adapt to local conditions, and this may affect their growth and reproductivity performance, which may subsequently affect the quality of the produced CS DOCs. Temperature, in particular, has been shown to significantly affect the reproduction performance of chickens (24).

Breeding farms interviewed had high levels of biosecurity protocols, with PS producers vaccinating DOCs against Newcastle disease, infectious bronchitis, infectious bursal disease, and Marek’s disease, as mandated by Veterinary Authorities (25). All CS producers reported vaccinating DOCs against Mareks, while only some extended vaccination against Newcastle disease, infectious bronchitis, and infectious bursal disease, which may serve as a marketing strategy as reflected by preference of their DOCs by farmers who participated in the current study. Vaccination against common poultry diseases is a critical component of disease prevention and control (26, 27), and has been shown to reduce the need for antibiotic use in livestock production (28). Hatching eggs were rigorously graded, with only those meeting strict quality criteria of cleanliness, size, shape, and shell integrity selected for production.

We found that DOCs breeding and production in Kenya are highly regulated. The Directorate of Veterinary Services has established guidelines to ensure the breeding and production of high-quality DOCs (25) with compliance monitored through annual audits of breeding farms and hatcheries, focusing on biosecurity, animal welfare, and routine *Salmonella* surveillance (25). However, regulatory oversight and enforcement during the distribution phase (hatchery-to-broiler farm) remains limited. Poor animal welfare and health practices were observed, including the transportation of DOCs over long distances, sometimes lasting up to 3 days, particularly when imported from neighboring countries or when distributors lacked an immediate market. Transport conditions were often suboptimal, with DOCs transported via public buses or motorbikes, exposing them to environmental stressors such as dust, rain, temperature fluctuations, and sunlight, and human contact and/or co-transportation with other goods. Prolonged transportation under such conditions has been associated with reduced DOCs viability, compromised immunity, lower body weight and feed intake, and increased mortality. Strengthening regulatory enforcement and monitoring across the distribution chain is thus needed to improve DOCs welfare and productivity outcomes.

Our analysis of the distribution chain identified multiple stakeholders, with veterinary drug stores playing a central role in the supply chain, connecting hatcheries and farmers. A recent study reported that 95.4% of farmers could access these stores within an hour using motorized transport (29), this close proximity highlights their critical function in providing animal health services. Despite the apparent role in antimicrobial stewardship, a significant proportion (3.5%) of drug stores in our study administered antibiotics to DOCs while in-store, and over half recommended antibiotic use to broiler farmers at the point of sale (“antibiotic starter packs”). Such practices expose apparently healthy DOCs to antimicrobials at the onset of production, potentially selecting for resistant bacterial strains early in the production cycle and facilitating their dissemination within flocks and the surrounding environment. Distributors cited various reasons for recommending antibiotics, including prevention of potential disease outbreaks, meeting farmer expectations, increasing sales, “boosting DOCs immunity,” and relieving stress. Such non-therapeutic antibiotic use underscores the tension between economic benefits and ethical dilemmas where the drive to minimize production losses often outweighs long-term public health risks. Comparable findings have been reported in Uganda, where poultry farmers viewed antibiotics as indispensable to maintaining flock health, preventing growth setbacks, and sustaining profitability (30). In our study, the most sold antibiotic starter pack contained four active ingredients—erythromycin, streptomycin, oxytetracycline, and colistin—administered as a “welcome cocktail” for DOCs. The use of such multi-drug formulations may contribute to early selection and persistence of antimicrobial resistant bacteria on farms. Studies investigating antimicrobial resistant carriage in DOCs or across the production and distribution channels are critically needed yet largely absent in most low- and middle-income country settings. It is possible that antibiotic starter packs, often containing multiple antibiotics, contribute to disproportionately high antimicrobial resistant levels in broiler production systems in low- and middle-income countries. However, empirical evidence to delineate this contribution is lacking.

Most of the findings in our study are derived from qualitative data and estimated proportions obtained through focus group discussions and key informant interviews. This is an inherent limitation of exploratory qualitative studies. However, we interviewed various stakeholders across the supply chain to allow for triangulation and minimization of errors. Our study mapped patterns and practices in the main poultry production areas, it is possible that some supply chains operating in other regions were not represented. Future studies should therefore adopt a broader geographical scope to capture the full diversity of distribution networks. We do not report quantitative data as these were unavailable, particularly from hatcheries, where such information was considered trade-sensitive and thus only reported as estimates. Future research should incorporate quantitative mapping and systematic data collection to complement these findings and provide a more comprehensive understanding of antimicrobial use and related practices across the poultry sector. Selection of participants in this study was non-random and relied on contacts provided by government officials and other stakeholders through a snowballing approach; as such, this may have introduced selection bias.

## Conclusion

Our study offers a new perspective by systematically linking the structure and functioning of the DOC supply chain with disease management and antibiotic use practices—an area that has received limited empirical attention in Kenya and comparable settings—thereby revealing critical structural, regulatory, and biosecurity gaps with direct implications for animal health, productivity, and antimicrobial stewardship. While DOCs breeding and production are relatively well regulated, the distribution and handling stages remain poorly governed, enabling practices that compromise bird welfare and encourage indiscriminate antibiotic use.

The central role of veterinary drug stores in this network positions them as both essential access points for farmers and potential hotspots for inappropriate antimicrobial dispensing and advice. For example, the widespread promotion of “antibiotic starter packs” for DOCs often containing multiple drug classes, including critically important antimicrobials such as colistin highlights an urgent need for targeted stewardship interventions. Strengthened regulation, improved welfare standards, and targeted training for distributors, farmers, and regulators are essential to enhance DOCs quality, promote responsible antibiotic use, and ensure sustainable poultry production. Further studies are needed to quantify DOCs-associated antibiotic use and its role in AMR emergence and transmission.

## Data Availability

The raw data supporting the conclusions of this article will be made available by the authors, without undue reservation.
